# Enhancing Preclinical Training for Removable Partial Dentures Through Participatory 3D Simulation: Development and Usability Study

**DOI:** 10.2196/71743

**Published:** 2025-09-19

**Authors:** Yikchi Siu, Hefei Bai, Jung-Min Yoon, Hongqiang Ye, Yunsong Liu, Yongsheng Zhou

**Affiliations:** 1 Department of Prosthodontics, Peking University School and Hospital of Stomatology, National Center of Stomatology, National Clinical Research Center for Oral Diseases, National Engineering Research Center of Oral Biomaterials and Digital Medical Devices, No. 22 Zhongguancun South Avenue, Haidian District, Beijing, China, 86 13066994235

**Keywords:** removable partial dentures, preclinical training, simulation, 3D, participatory

## Abstract

**Background:**

The integration of digital technology in dental education has been recognized for its potential to address the challenges in training removable partial denture (RPD) design. RPD framework design is crucial to long-term success in the treatment of dentition defects, but traditional training methods often fall short of adequately preparing students for real-world applications.

**Objective:**

This study aimed to evaluate the efficacy of a 3D simulation–based preclinical training software for RPDs in enhancing learning outcomes among first-year stomatology master’s students, while also assessing user perceptions among students and faculty.

**Methods:**

RTS (Yikchi Siu) is a preclinical training software that simulates the clinical process of treating patients with partial edentulism. In this study, 26 newly enrolled master’s degree students in stomatology who volunteered to participate were randomly divided into a control group (n=13) and a training group (n=13). The training group used the RTS for 2 credit hours (90 min) of self-study, while the control group received theoretical lessons and case practice from an instructor. After 2 hours, both groups completed the theoretical knowledge and drawing tests for RPD simultaneously. Test results were evaluated and graded by 2 experts in prosthodontics. Both users and teachers filled out a questionnaire afterward about their training experience.

**Results:**

Participants in the training group obtained better final grades compared to controls (theoretical test: 88.8, SD 2.3; 85.7, SD 3.3, respectively; *P*=.01; drawing test: 89.8, SD 4.5; 85.1, SD 4.3, respectively; *P*=.01). The training group had a shorter completion time in the drawing test (12.6, SD 19 min; 17.7, SD 3 min, respectively; *P*<.001) but there were no significant differences in the completion times in the theoretical test (23.2, SD 2.2 min; 24.9, SD 2.8 min, respectively; *P*=.14). Students and faculty generally had a favorable opinion of the RTS.

**Conclusions:**

The effectiveness of the RTS for newly enrolled master’s degree students in stomatology to understand and apply their knowledge of RPD framework design was validated; the system was well received by both students and faculty members, who reported that it improved the effectiveness and convenience of teaching.

## Introduction

As life expectancy and the size of the aging population continue to increase, so does the prevalence of patients with partial edentulism [1]. Removable partial dentures (RPDs) are versatile and cost-effective alternatives to fixed and implant restorations, and thus an increasing number of patients are choosing them to restore their dentition [2-4].

In the conventional training paradigm, students predominantly use 2D graphic representations and textual exercises to design RPD frameworks [5]. However, this is very different from how it is done in actual clinical practice [6,7]. In fact, RPD framework design is an ongoing challenge within the context of prosthodontics training [8]. This is largely attributable to the intricate nature of RPD components and the considerable diversity of design proposals [9,10]. The design process requires the synthesis of theoretical knowledge and clinical practice; that is, a high degree of clinical decision-making ability is necessary [7,11].

The advent of simulation technology in dentistry has led to immersive training systems including some based on force feedback and mixed reality [12]. However, there is a lack of studies on nonimmersive simulation approaches. In this context, to enhance the efficiency of training in the design of RPD frameworks, the use of training systems that incorporate 3D casts has increasingly been recognized as an innovative and reliable approach [13]. One reason for this is the popularity of digital RPD design software, which enables users to export 3D casts in standard tessellation language format for use in training. The use of such casts facilitates a more comprehensive understanding of the design principles and components of the RPD framework [14-16]. A substantial body of evidence indicates that integrating such systems into teaching curricula can significantly enhance student interest and learning outcomes as well as the efficacy of teaching [5,16,17]. This study describes novel 3D RPD simulation software designed for preclinical training in RPD framework design and provides a preliminary evaluation of its efficacy. We hypothesized that RTS (Yikchi Siu) would improve the test scores of RPD design and reduce completion time compared to traditional training methods.

## Methods

### RPD Training System

**Figure 1.**
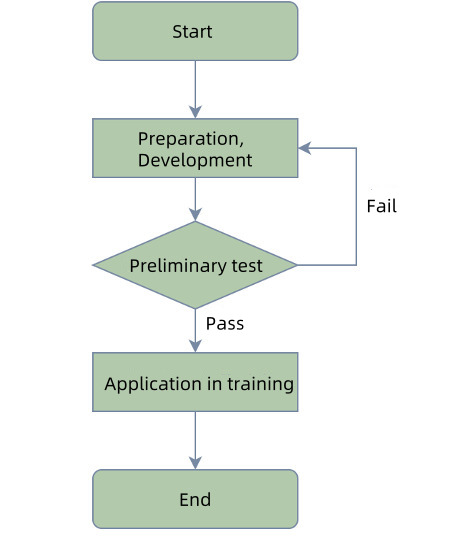
Flowchart of RTS development.

RTS is a participatory training program developed to enhance the RPD design skills of users without preclinical training. We designed and tested it in 3 phases: software preparation and development, preliminary tests, and application in training (Figure 1). During the development phase (August 2023 to July 2024), a prototype was prepared, designed, and developed. In the test phase (August 2024), 5 students (3 master’s students and 1 doctoral student) enrolled at the Peking University School of Stomatology were recruited to test the system, and based on the outcomes, a panel of experts was asked to suggest modifications to the RTS; the system was modified accordingly. Finally, in the training phase (September 2024 to October 2024), the RTS was introduced to 26 newly enrolled postgraduate students who had not received preclinical training, and the system was quantitatively and qualitatively assessed by analyzing their test scores and feelings about its use. The study design followed the recommended framework for software development [18] and the criteria for the validation process [19].

RTS was developed using Visual Studio Code and is a standalone program for Windows 10 (Microsoft Corporation). The software includes 3D digital casts created from clinical plaster casts from 19 dental patients. These cases were carefully selected and prepared by the software developers and then reviewed and approved for relevance and accuracy by 2 prosthodontics experts. The digital casts were created using the 3Shape cast scanner, replicating real-world clinical conditions.

**Figure 2.**
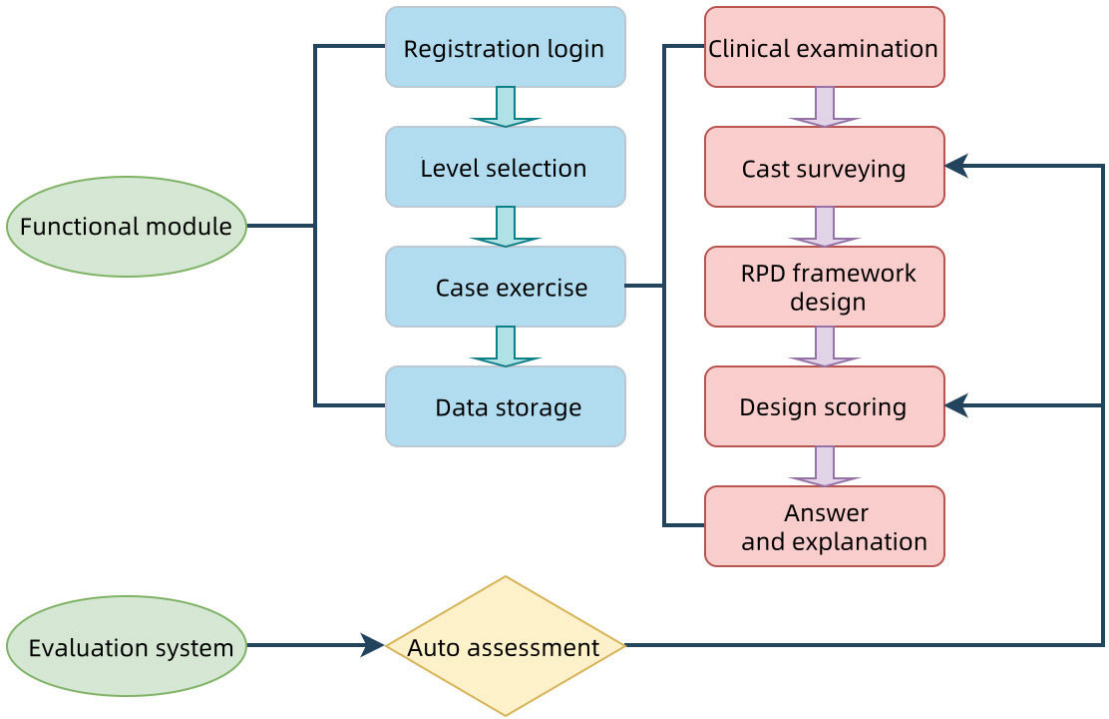
Framework of RTS. RPD: removable partial denture.

The training content is divided into 5 topics: clinical examination, cast surveying, RPD framework design, design scoring, and answer and explanation (Figure 2). The RTS includes a functional module and an evaluation system. The former facilitates user engagement, and the latter involves assessment, design, and evaluation of user outcomes. The auto assessment component quantitatively assesses user performance, providing a comprehensive educational tool for preclinical dental training. Users view the 3D cast and perform digital surveying with mouse clicks. If the surveying perspective is incorrect, an error message appears, and the user cannot proceed with the RPD framework design until the cast is oriented correctly. Once the surveying angle is correct, a confirmation message is displayed. Users then design an RPD framework blueprint based on the clinical examination and cast survey data.

**Figure 3.**
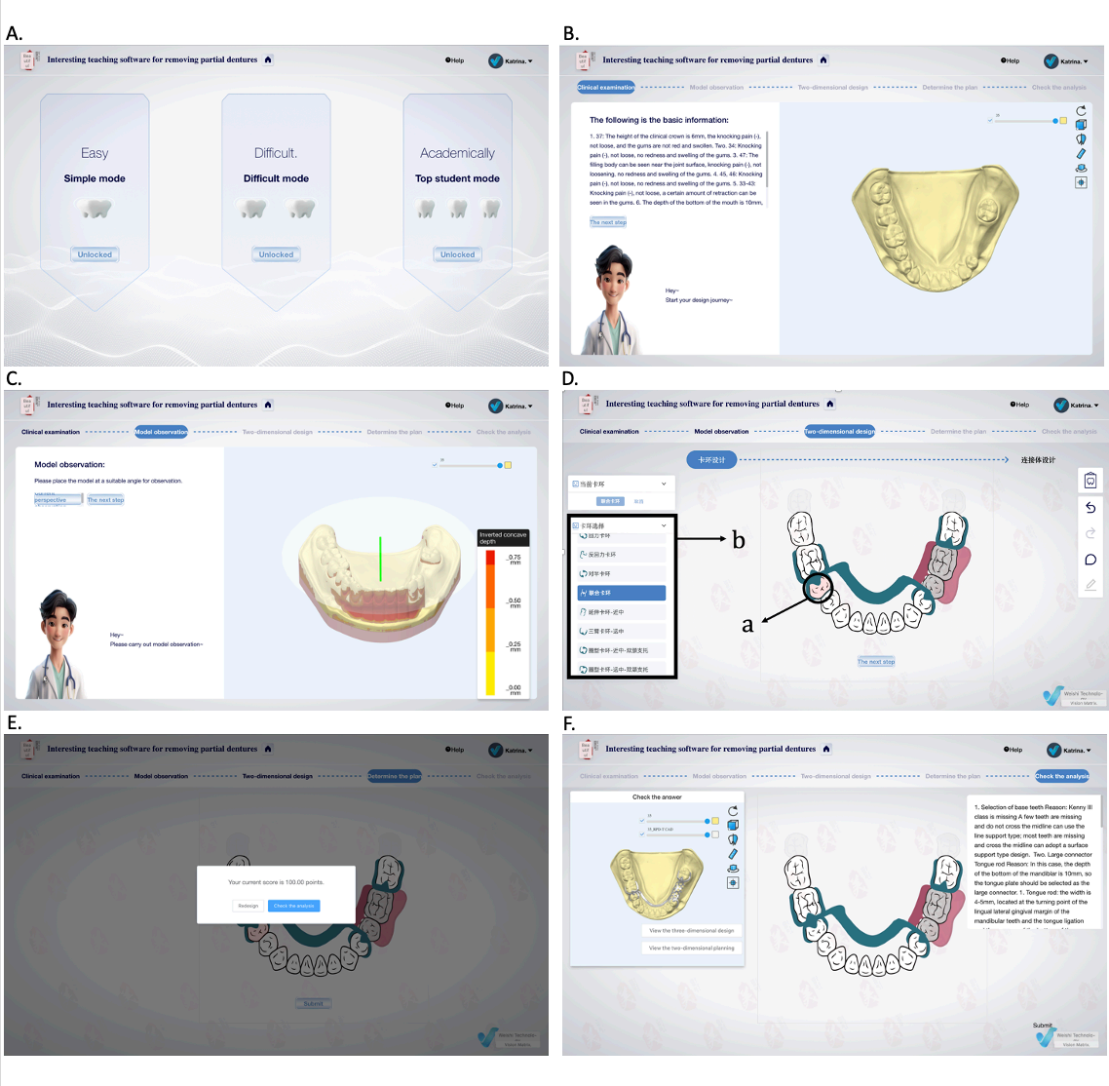
Screenshots from the RTS.

After completing the design, users submit it for automatic scoring by the RTS evaluation system, which provides immediate feedback, including a standardized design for comparison. Faculty members can also review and manually grade the designs in the background (Figure 3). Figure 3 shows an example of a Kennedy classification III case exercise. The user logs on to the webpage and selects an available training mode (Figure 3A). Each mode contains 6 to 7 practice cases, all of which must be successfully completed before the next mode will be unlocked. The user clicks and rotates the 3D cast of a dental defect to view it, determine the Kennedy type, and obtain information about the intraoral clinical examination (Figure 3B). Then the user adjusts the appropriate viewing angle to survey the inverted concave area (Figure 3C). Next, the user designs the framework for RPD (a: red indicates the selected tooth as an abutment; b: selection of an appropriate clasp design), including features such as abutment teeth, clasps, rests, and major and minor connectors, to complete the design for the case (Figure 3D). The design is submitted and the system automatically scores it (Figure 3E). A system score of 60 and above will display the standardized design plan and an explanation; otherwise, the case must be designed again (Figure 3F). To start the RPD case practice, users must open the provided URL [20], register, and log in. Then the system automatically saves their practice records.

### Application

#### Sample Size Calculation

A pilot study will be conducted with 5 newly admitted graduate students per group. Sample sizes will be calculated independently for both primary end points (drawing test scores and test time), with the larger of the 2 values being adopted to guarantee adequate power for all outcome assessments. Using pilot data of drawing test time (control group: 17.7, SD 2.7 min; training group: 12.6, SD 1.8 min) with α=.05 (2-tailed) and 80% power, the minimum sample size was 7 per group (total 14). Considering educational intervention complexity, we enrolled 13 per group (total 26).

#### Quantitative Assessment

For quantitative assessment, 26 newly enrolled graduate students who had not received RPD preclinical training volunteered to assess the system. In addition, the students were recruited and randomly allocated via sealed envelope drawing into 2 groups, the control group (n=13) and the training group (n=13).

Both groups first underwent a preclinical training course for RPDs. The instructor provided an explanation of the functionality of the software to ensure that each participant had the requisite knowledge and proficiency to use it. Then the training group was instructed to use the RTS to learn and practice the RPD framework design independently. By contrast, the graduate students in the control group were required to learn the theoretical aspects of RPD and complete the associated classroom exercises under the guidance of an instructor. During the period, the control group was not permitted to use RTS for learning purposes. At the conclusion of the experiment, both groups were administered a quiz assessing their theoretical knowledge of the RPD framework design and their design ability. The test included 10 clinical cases of dentition defects which were developed by 2 experts in prosthodontics, including 3 cases each of Kennedy Class I and II, and 2 cases each of Class III and IV. The assessment was conducted by 2 prosthodontic experts using a 100-point grading scale established based on RPD design principles and requirements from textbooks [21,22] and the National Dental Practitioner Training Manual (Table 1). The final scores were calculated as the mean values of the ratings given by 2 independent experts for each student group’s assessments.

**Table 1.** Removable partial denture design drawing evaluation rubric.

Category (subcriteria)	Maximum score	Remarks
I. Basic design principle (20)		
Fulcrum line design	6	Consistency with task description
Force distribution	6	Balanced occlusal loading
Antirotation or sinking measures	4	Indirect retainers or rests
Lever arm	2	Minimize cantilever
Stress breaker	2	If applicable
II. Component design (20)		
Direct retainers	10	Clasp type selection
Indirect retainers	5	Position rationality
Major connectors	5	Type selection
III. Clinical applicability (10)		
Kennedy classification	5	Correct I-IV
Esthetic considerations	2.5	Consistency with case description
Functional considerations	2.5	Consistency with case description
IV. Drawing (50)		
Missing tooth position	20	Accurate identification and correct marking
Color	10	Blue metal components and red resin bases
Neatness	10	Neat and accurate
Component drawing	20	Component positioning and drawing Minor connector spacing and drawing Finish line

#### Qualitative Assessment

To evaluate the subjective efficacy of the RTS, a questionnaire was administered to students and staff to assess user experiences as well as instructor and student satisfaction with the system (Multimedia Appendix 1). The questionnaire included 10 questions, with respondents indicating their level of satisfaction or agreement with statements ranked on a 5-point Likert item (1=strongly dissatisfied or strongly disagree to 5=strongly satisfied or strongly agree) [12]. The questionnaire includes 6 student-specific questions and 4 teacher-specific questions. An open-ended question was also included to elicit feedback on potential shortcomings.

#### Statistical Analysis

This study used a combination of descriptive and inferential statistics to evaluate the scores and time spent on theoretical and drawing tests. First, all data were summarized descriptively using means and SD to illustrate the central tendency and dispersion of each group. Normality was assessed using the Shapiro-Wilk test, which was prioritized for its robustness with small sample sizes (n<50). Homogeneity of variance was evaluated via *F* tests to validate the assumptions for subsequent parametric tests. Results confirmed that all 4 datasets met both normality and homoscedasticity assumptions. Independent samples *t* tests were used for 2-group analyses, reporting *t* values, degrees of freedom (*df*), and *P* values. Mann-Whitney *U* tests were concurrently performed to ensure robustness, reporting *U*, *z*, and *P* values. Effect sizes were quantified using Cohen *d* to complement statistical significance. To examine linear agreement between 2 experts’ scores, interrater reliability was assessed through Pearson correlation coefficient and intraclass correlation coefficient (ICC) under a 2-way random-effects absolute agreement model, accounting for both systematic differences and random error between raters. Statistical evaluation was performed using the SPSS analysis program (IBM SPSS Statistics 26.0, IBM Corp). GraphPad PRISM 10.0 software (GraphPad Software) was used to create graphs.

### Ethical Considerations

This study was approved by the ethics committee of Peking University School and Hospital of Stomatology (PKUSSIRB-202273003-免). Participants understood the study details and their rights, including their right to withdraw at any point without consequences. Participants’ privacy is secured in all written and published data arising from this study. Names of participants or other identifying information are not and will not be used in reports or published papers.    

## Results

### Quantitative Assessment: Test Scores

**Figure 4.**
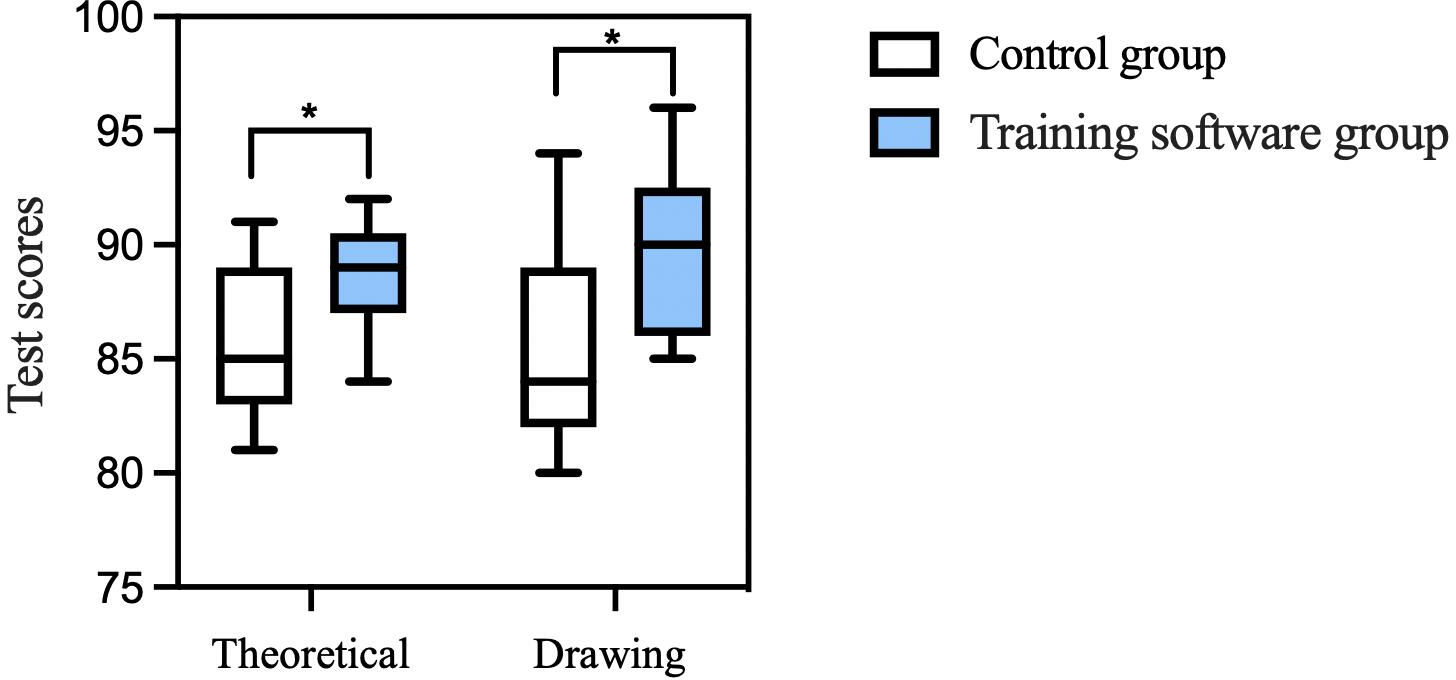
Comparison of test scores between the control and training groups. *P<.05.

As shown in Figure 4, the theoretical test score in the training group was 88.8 (SD 2.3), while the drawing test score was 89.8 (SD 4.5). These values were 85.7 (SD 3.3) and 85.1 (SD 4.3), respectively, in the control group. Thus, the training group had significantly better scores for both tests (*P*=.01). Moreover, the Mann-Whitney *U* test was further applied for evaluation, and the *P* value remained less than .05, both groups with a large effect size (Cohen *d* = 1.1). The nonparametric test confirmed this result on the theoretical test (*U*=33.0, *z*=–2.5, *P*=.01) and the drawing test (*U*=33.0, *z*=–2.5, *P*=.01) (Figure 4). A strong positive correlation was observed between the raters’ evaluations (Pearson *r*=0.70, *P*<.001; ICC=0.84, 95% CI 0.7-0.9).  

### Quantitative Assessment: Test Time

**Figure 5.**
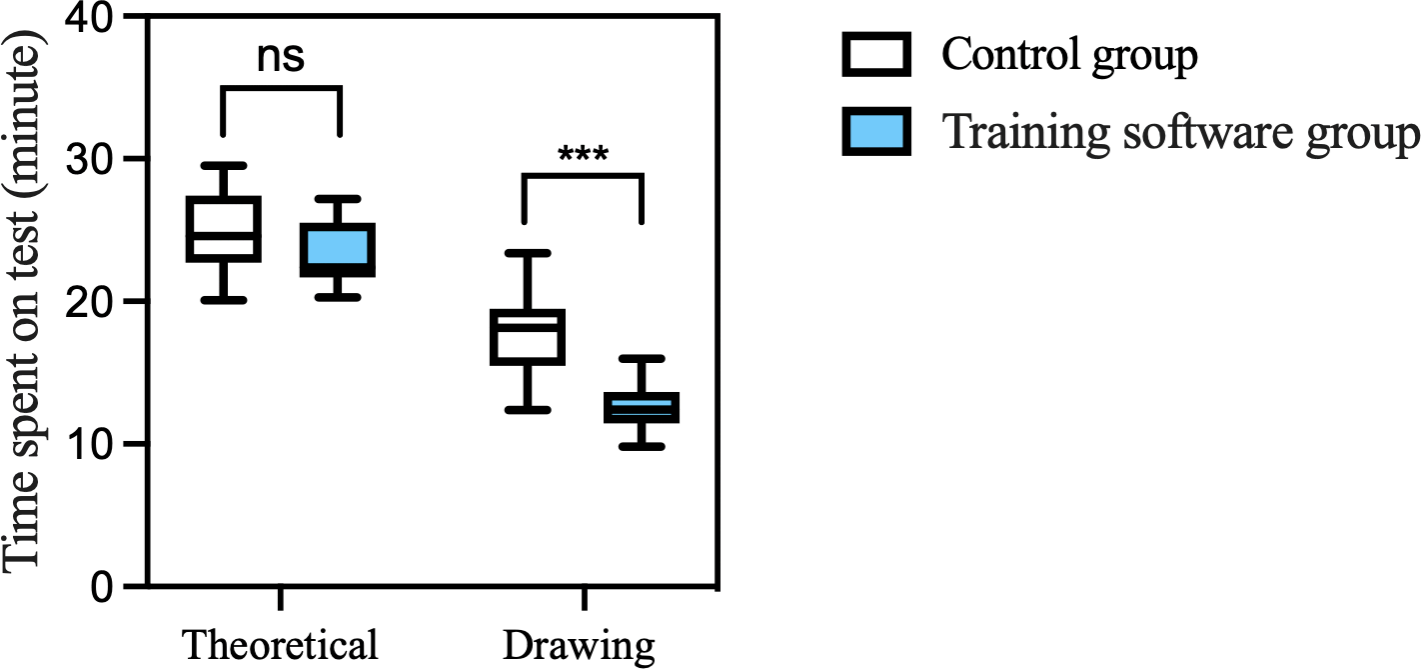
Time spent on tests. ***P<.001, ns: not significant.

As shown in Figure 5, the mean completion times were 23.2 (SD 2.2) minutes in the training group and 24.9 (SD 2.8) minutes in the control group for the theoretical test; they were 12.6 (SD 1.9) minutes and 17.7 (SD 3) minutes for the drawing test, respectively, with a significant difference in the latter test only (*P*<.001).

### Response to Questionnaire

As shown in Table 2, users found the RPD framework design to be easier and more accessible when using the RTS. In addition, all users indicated that the program was convenient and straightforward to use. Users indicated a greater preference for the RTS over traditional training methods and expressed great satisfaction with the responsive features. Faculty members also had positive views, reporting that the system significantly reduced preparation time for teaching and improved teaching effectiveness.

**Table 2.** Responses of the training group (n=13) to the questionnaire.

Questionnaire item	Very dissatisfied or strongly disagree, n (%), 1 Point	Dissatisfied or disagree, n (%), 2 Points	Neutral, n (%), 3 Points	Satisfied or agree, n (%), 4 Points	Very satisfied or strongly agree, n (%), 5 Points	Mean (SD)
The software program encourages users to practice in designing RPD^a^ frameworks.	0 (0)	0 (0)	0 (0)	1 (8)	12 (92)	4.9 (0.3)
The software program is highly responsive and allows for real-time feedback.	0 (0)	0 (0)	0 (0)	2 (15)	11 (85)	4.8 (0.4)
The software program is easy to use.	0 (0)	0 (0)	0 (0)	0 (0)	13 (100)	5 (0)
The software program effectively helps users to learn and practice RPD design, enhancing decision-making and critical skills.	0 (0)	0 (0)	1 (8)	1 (8)	11 (85)	4.8 (0.6)
The software program allows users to learn more about surveying 3D casts and digitally drawing designs.	0 (0)	0 (0)	0 (0)	0 (0)	13 (100)	5 (0)
I would like to use the RTS for skills training in the future.	0 (0)	0 (0)	1 (8)	3 (23)	9 (69)	4.6 (0.6)

a RPD: removable partial denture.

## Discussion

### Principal Findings

Based on the above experiments, it can be concluded that the RTS significantly improved both theoretical and drawing test scores related to RPD design among newly enrolled graduate students. Moreover, the system substantially reduced the time required for students to complete RPD design drawings. The positive feedback from both students and faculty members further suggests that RTS shows great promise as a preclinical training software for RPD design.

With the accelerated development of digital technology and the emergence of new educational models, online responsive training methods are playing an increasingly pivotal role in the field of medical education [23-25]. Such methods have notable benefits in terms of reduced teaching costs, providing an immersive experience, enabling resource sharing, and offering personalized learning and feedback, to name a few [26,27]. In the field of dental education, RPD design is a key ongoing challenge and area of focus. Students frequently have difficulty grasping the concepts related to RPD components and lack access to case-based instruction [5,28]. This is due to the inherent limitations of traditional teaching methods, which often lack the necessary casts and RPD frameworks needed for effective preclinical training, as well as the inability to practice on clinical cases [16,29,30]. The AiDental system was previously designed to teach RPD design, and has demonstrated effective teaching outcomes for RPD design instruction in dental education. However, Mahrous et al [31] have revealed 2 persistent limitations in the study: a suboptimal user interface design that may reduce learner engagement and insufficient analytical feedback on student performance. The design of Musawi et al [32] contains a specific clinical RPD process but uses 2D graphics, which lack a sense of immersion and interactivity. The RTS overcomes these limitations.

Using RTS, students can treat dentition defects and design RPD frameworks in a simulated clinical setting. The absence of a surveyor or plaster casts during the surveying and design processes ensures that the training content is more closely aligned with the clinical environment, thereby creating a more realistic training experience. This approach allows the trainer to integrate theoretical knowledge with clinical practice effectively, facilitating a smoother transition from theory to practice.

Regarding its efficacy, the training group achieved better results than did controls in both the theory and drawing tests. This means that the program helped users to understand and master RPD design theory better, increasing the design speed. This may be because the program allows users to create personalized study plans based on their individual learning abilities and mastery levels, which significantly improves learning efficiency.

There were no statistically significant differences in the times taken by the 2 groups to perform the theory test, while the training group spent significantly less time completing the drawing test. This indicates that the program facilitated a more comprehensive understanding, making students more proficient in RPD design. The immediate feedback on, and comprehensive analysis of, incorrect responses provides a rapid-response mechanism for challenging content, enabling users to identify knowledge gaps accurately in a time-efficient manner. Moreover, the case practice and personalized guidance module of the program facilitates one-on-one training for students, in contrast with the traditional classroom setting.

Finally, both students and teachers viewed the software program positively. Students gave high ratings for user-friendliness, indicating that it allowed them to grasp essential functions quickly. They expressed a high level of interest in the RTS and perceived it to be more engaging and participatory than conventional paper-based RPD design. In addition, users were more inclined to dedicate time to the RTS to gain insight into RPD design. In fact, all of the users (n=13) indicated that the program allowed them to learn how to survey casts digitally, which is particularly beneficial in the context of the increasing use of CAD (Computer-Aided Design) software and 3D casting. Furthermore, users indicated a desire to do more digital training over traditional training in the future.

Teachers reported that the system notably reduced the time required for lesson planning. Instructor preparation time decreased from 90 minutes (traditional methods) to 45 minutes using RTS. They also felt that it made teaching easier and resulted in improved outcomes. Finally, they indicated a willingness to integrate such systems into future lesson plans as a potential option for self-study and after-school practice with students.

While the current system demonstrates promising educational outcomes, several refinements could further enhance its efficacy. Incorporating advanced haptic feedback technology could improve students’ understanding of RPD biomechanics by simulating real-world force distribution during clasp design and framework adaptation. Recent studies have shown that force-feedback systems significantly enhance skill acquisition in preclinical dental training, particularly in prosthodontic procedures requiring precise tactile sensitivity [33,34]. In addition, integrating augmented reality for surveying and undercut analysis could bridge the gap between simulation and clinical application, as augmented reality has proven effective in improving spatial awareness in dental education [35]. To maximize impact, future iterations should explore embedding this tool into official dental curricula as a standardized preclinical training module. Structured integration, as seen with successful simulation platforms in medical education [23], would facilitate widespread adoption. Multi-institutional collaborations could also validate their scalability, similar to the collaborative frameworks used in the Association for Dental Education in Europe guidelines for simulation-based training [36]. By addressing these refinements and curricular integration pathways, the system has the potential to become a benchmark for RPD education.

### Limitations and Future Work

The limitations of this study include the relatively small sample size, the recruitment of participants from a single school, and the absence of a randomization process. In addition, there may have been unidentified differences in the intrinsic and current learning abilities of the students. To enhance the rigor and reliability of the study, a randomization process could be used to balance the groups and mitigate the impact of confounding variables, thereby yielding higher-quality evidence. There is a paucity of data on the efficacy of such training modules for dental students. Consequently, future studies should collect more data to validate the long-term effectiveness and clinical transferability of these methods in real-world preclinical training settings, thereby facilitating their broader adoption [37,38]. Due to the inability to perform real-time adjustments for nonstandard anatomical structures, the model surveying function in RTS demonstrates poorer adaptability for complex cases. Manual operation with traditional surveyors enhances spatial awareness and biomechanical understanding, which digital tools may not fully replicate. So, it is important to note that the surveying function in the RTS cannot replace the use of an actual physical surveyor for preclinical training. This feature is designed solely to provide students with preliminary practice in understanding model survey angles. Comprehensive mastery of model surveying techniques still requires training with a physical surveyor.

Simulation can be an indispensable tool for training students so that they develop the necessary skills for authentic real-world scenarios. Our results show that the RTS effectively enhances the efficacy of RPD design instruction. It offers a flexible training module that can be adapted to evolving trends in dental education.

### Conclusions

The novel RTS effectively improves the teaching and practice of RPD design. This study has described the system and offered a preliminary validation of its effectiveness. The system was viewed positively by both students and teaching staff. It enhanced the design competence of students not previously exposed to standardized preservice training in prosthodontics. It also made teaching easier and led to better teaching and learning outcomes. Thus, it can be considered an effective tool for teaching and learning RPD design.

## Supplementary material

10.2196/71743 Multimedia Appendix 1 User satisfaction questionnaire for RTS.

## Abbreviations

ICC: intraclass correlation coefficient
RPD: removable partial denture
