# Supplementary material for: Enhancing Preclinical Training for Removable Partial Dentures Through Participatory 3D Simulation: Development and Usability Study
Source: JMIR Med Educ. 2025 Sep 19;11:e71743. doi: 10.2196/71743 (PMC12454677; doi:10.2196/71743)
Supplement: Multimedia Appendix 1 [file mededu-v11-e71743-s001.docx]

User Satisfaction Survey for RTS

Please indicate your level of agreement or satisfaction with each statement by selecting the appropriate number (1–5), where:

- 1 = Strongly dissatisfied / Strongly disagree
- 2 = Dissatisfied / Disagree
- 3 = Neutral
- 4 = Satisfied / Agree
- 5 = Strongly satisfied / Strongly agree

| **No.** | **Statement** | **1** | **2** | **3** | **4** | **5** |
| --- | --- | --- | --- | --- | --- | --- |
| 1. | The software program encourages users to practice in designing RPD frameworks. | ○ | ○ | ○ | ○ | ○ |
| 2. | The software program is highly interactive and allows for real-time feedback. | ○ | ○ | ○ | ○ | ○ |
| 3. | The software program is highly interactive and allows for real-time feedback. | ○ | ○ | ○ | ○ | ○ |
| 4. | The software program effectively helps users to learn and practice RPD design, enhancing decision-making and critical skills. | ○ | ○ | ○ | ○ | ○ |
| 5. | The software program allows user to learn more about surveying 3D casts and digitally drawing designs. | ○ | ○ | ○ | ○ | ○ |
| 6. | I would like to use the RTS for skills training in the future. | ○ | ○ | ○ | ○ | ○ |

**Additional Feedback (For Teachers Only)**

| **No.** | **Statement** | **1** | **2** | **3** | **4** | **5** |
| --- | --- | --- | --- | --- | --- | --- |
| 1. | Using RTS for instruction helps students better master RPD framework design. | ○ | ○ | ○ | ○ | ○ |
| 2. | Students demonstrate higher engagement when RTS is used in teaching. | ○ | ○ | ○ | ○ | ○ |
| 3. | RTS makes pre-class lesson preparation more convenient. | ○ | ○ | ○ | ○ | ○ |
| 4. | **Approximately how much time does RTS save you in lesson preparation?** (Please specify): _______________ |  |  |  |  |  |
